# Multi-Institutional CT Scan-Based Radiomics for Predicting Tumor PD-L1 Expression in Patients with Advanced and Limited Non-Small Cell Lung Cancer

**DOI:** 10.3390/cancers18040552

**Published:** 2026-02-08

**Authors:** Ralph Saber, Marion Tonneau, Olivier Salko, Moishe Liberman, Julie Malo, Arielle Elkrief, Simon Turcotte, Nicole Bouchard, Philippe Joubert, Samuel Kadoury, Bertrand Routy

**Affiliations:** 1 MedICAL Laboratory, Polytechnique Montreal, Montreal, QC H3T 1J4, Canada; ralph.saber@polymtl.ca (R.S.); samuel.kadoury@polymtl.ca (S.K.); 2 Research Center of the Hospital Center of the University of Montreal, Montreal, QC H2X 0A9, Canada; marion.tonneau.chum@ssss.gouv.qc.ca (M.T.); olivier.salko@umontreal.ca (O.S.); moishe.liberman@umontreal.ca (M.L.); julie.malo.chum@ssss.gouv.qc.ca (J.M.); arielle.elkrief.med@ssss.gouv.qc.ca (A.E.); simon.turcotte.med@ssss.gouv.qc.ca (S.T.); 3 Department of Medicine, Lille University, 59020 Lille, France; 4 Hospital Center of the University of Sherbrooke, Sherbrooke, QC J1G 1B1, Canada; nicole.bouchard@usherbrooke.ca; 5 University Institute of Cardiology and Pneumology of Quebec, Laval University, Quebec City, QC G1V 0A6, Canada; philippe.joubert@criucpq.ulaval.ca

**Keywords:** immune checkpoint blockade, programmed death-ligand 1, noninvasive biomarker, non-small cell lung cancer, radiomics

## Abstract

Background/Objectives: Immune checkpoint inhibitors (ICIs) have revolutionized the treatment landscape of advanced non-small cell lung cancer (NSCLC), yet 70% of patients experience disease progression, underscoring the critical need for predictive biomarkers. Programmed death-ligand 1 (PD-L1) expression remains the most adopted biomarker for ICIs. With the emergence of machine learning, the development of radiomics algorithms based on CT scan images has demonstrated potential as a novel addition to the biomarker landscape in oncology. In this study, we aimed to develop a non-invasive surrogate of PD-L1 expression (rad-PDL1) derived from computed tomography (CT) scan imaging and compare its predictive value to pathological assessments. Furthermore, we evaluated its generalizability across advanced and limited-stage NSCLC. Methods: Radiomics features extracted from pretreatment CT were analyzed using a self-training pipeline that incorporated the feature tokenizer Transformer model to classify tumors as high vs. low PD-L1 expression. We included 482 advanced NSCLC patients treated with ICIs across three medical centers who were divided into training and hold-out validation sets. The algorithm was then further validated in an independent cohort of 51 patients with limited NSCLC treated with neoadjuvant ICI and chemotherapy. Results: Our pipeline demonstrated strong predictive performance in primary and independent validation (AUC = 0.75 and 0.68, accuracy = 0.73 and 0.69, respectively), highlighting its generalizability and adaptability to various disease stages. Kaplan–Meier curves revealed a longer progression-free survival for patients in the high rad-PDL1. Conclusions: These results demonstrate the feasibility of a CT-based radiomic surrogate of PD-L1 expression, showing partial generalization to an independent neoadjuvant cohort, while highlighting the need for larger prospective multi-site validation before clinical implementation.

## Simple Summary

Immunotherapy has significantly improved the treatment of lung cancer; however, a large proportion of patients do not experience durable benefit, making it challenging to identify those most likely to respond. At present, treatment decisions rely on the assessment of tumor tissue, which requires invasive biopsies and may not fully capture tumor heterogeneity. In this study, we investigated whether information routinely available from computed tomography (CT) scans could be used to estimate a key immunotherapy biomarker, programmed death-ligand 1 (PD-L1), without additional invasive procedures. Using artificial intelligence–based image analysis, we developed a CT-derived score associated with PD-L1 expression and clinical outcomes. We demonstrated that this approach is applicable across different stages of non-small cell lung cancer and treatment settings. These results indicate that, upon prospective validation, CT-based features may support more precise patient stratification and contribute to personalized immunotherapy strategies.

## 1. Introduction

Despite the paradigm shift instigated by the approval of immune checkpoint inhibitors (ICIs) for the treatment of multiple malignancies, lung cancer continues to be the leading cause of cancer-related mortality in Canada, accounting for 23% of cancer deaths in 2024 [1]. Inhibitors of programmed death-1 (PD-1) or its ligand (PD-L1), either as monotherapy or in combination with platinum-doublet represent the cornerstone of treatment for patients with advanced non-small cell lung cancer (NSCLC) and have recently expanded in the neo-adjuvant setting (NSCLC) [2,3,4]. Nevertheless, less than 50% of patients experience sustained clinical benefit from ICIs [5]. As a result, a considerable number of patients experience potential risks and toxicities associated with treatments that are not guided by the projected outcome, failing to halt or impede disease progression.

Currently, no single biomarker fully captures tumor biology and forecast potential resistance to ICIs [6]. Nevertheless, PD-L1 expression remains the most reliable and dictate treatment strategy for patients with advanced NSCLC without driver mutations [7,8]. The phase III KEYNOTE-024 study demonstrated that in advanced NSCLC patients with high PD-L1 expression (≥50%), pembrolizumab significantly extended the median progression-free survival (PFS) compared to chemotherapy (7.1 vs. 6.4 months). Moreover, pembrolizumab demonstrated superior efficacy in terms of 5-year overall survival (OS) compared to platinum-based chemotherapy, with OS rates of 31.9% and 16.3%, respectively [9]. Conversely, in the KEYNOTE 042 trial, patients with PD-L1 < 50% did not benefit from single-agent anti-PD-1. Therefore, following several positive trials with combination strategies involving anti-PD-1 +/− CTLA-4 with platinum doublet have become the standard of care for patients with PD-L1 expression < 50% [2,10,11]. Additionally, the integration of a combination of anti-PD-(L)1 and chemotherapy has become the standard of care for patients in the neo-adjuvant/perioperative settings for patients with limited-stage disease who are eligible for surgery [12,13,14,15]. These recent studies also suggest that patients with PD-L1 > 50% derive greater benefit from neo-adjuvant chemo-IO [14]. The rapid and accurate evaluation of PD-L1 expression remains paramount in patients with NSCLC. This is currently conducted on tumor biopsies using immunohistochemistry (IHC), which is associated with procedural limitations as well as several constraints, including intratumoral heterogeneity, variability between primary tumors and metastases, fluctuations in sample quality, and differences in staining protocols [16].

Conversely, computed tomography (CT) images are widely accessible in the context of NSCLC, as they are routinely acquired for initial diagnosis and ongoing monitoring [17]. The field of radiomics, which involves the extraction of hand-engineered features from medical images, has the potential to identify underlying genomic and proteomic properties that are imperceptible to the naked eye [18]. This approach enables the detection of subtle patterns within image voxels that may reflect molecular-level processes and tumor biology. Several studies have leveraged radiomics features and machine learning models to predict tumor infiltrating lymphocytes, namely cytotoxic T cells [19] and helper T cells [20] and the intratumoral expression of immune biomarkers [21] including PD-L1 expression [22,23]. Other works have focused on the prediction of genetic mutations for the KRAS [24] and EGFR [25,26,27] genes, as well as the tumor mutational burden [28]. Moreover, additional studies have sought to classify patients’ PFS at 6 months after treatment [29], since this clinical outcome was identified as one of the most reliable [30]. However, most radiomics studies are limited by single-center datasets, which lack variability in patients’ characteristics, data acquisition parameters, and the types of treatments administered. Moreover, these studies often lack subgroup analyses to validate the model’s external validity across diverse patient subpopulations, particularly in relation to disease stage and treatment strategy. Notably, there is a paucity of data regarding the model’s generalizability to NSCLC patients receiving neoadjuvant ICIs in combination with chemotherapy. They also provide minimal insight into the machine learning models employed, which are typically treated as “black boxes.” Ensuring that prediction models are generalizable across NSCLC patients receiving different treatments is essential for their clinical applicability and utility in diverse therapeutic settings. Consequently, there is a significant gap in multi-institutional research focused on developing reliable, noninvasive, and interpretable biomarkers of response to ICIs in NSCLC that can be generalized across various stages.

In this study, we present an AI-based pipeline trained on a multi-institutional dataset to predict PD-L1 expression in NSCLC patients treated with ICI. The pipeline leverages advanced deep learning techniques, including self-training to enhance its predictive accuracy and generalizability. We validate the pipeline on a hold-out testing set as well as in an independent cohort of limited-stage NSCLC patients treated with neoadjuvant ICI and chemotherapy. Additionally, we provide an interpretability analysis and evaluate the association of the probabilistic rad-PDL1 scores generated by the pipeline with patient PFS. Prior CT-based radiomics studies of PD-L1 expression have largely relied on fully supervised learning, single-center cohorts, and internal validation, limiting generalizability and scalability in the context of scarce pathological annotations. In contrast, the present study introduces a self-training framework that leverages unlabeled CT scans, is trained on multi-institutional data, and is evaluated on an independent neoadjuvant cohort. This design enables assessment of both data-efficient learning and preliminary cross-context generalization, addressing key limitations of prior radiomics approaches to PD-L1 prediction.

## 2. Materials and Methods

### 2.1. Study Population and Data Collection

**Figure 1:**
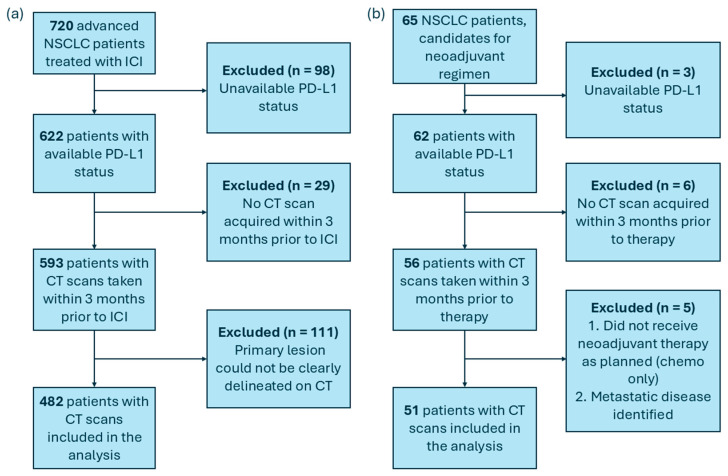
CONSORT-style diagram of patient selection in (**a**) the primary cohort and (**b**) the independent neoadjuvant cohort.

We conducted a retrospective analysis on 720 patients with advanced NSCLC. All subjects were treated between 2015 and 2022 using anti-PD-1 immunotherapy either in first-line or chemotherapy-refractory settings across three academic institutions: Centre Hospitalier de l’Université de Montréal (CHUM), Centre Hospitalier Universitaire de Sherbrooke (CHUS), and Institut Universitaire de Cardiologie et de Pneumologie de Québec–Université Laval (IUCPQ-UL). Moreover, we validated the AI-based pipeline on an independent cohort of 65 limited patients treated for NSCLC at CHUM using a neoadjuvant regimen combining chemotherapy with anti-PD-1 monoclonal antibody between 2019 and 2024. The inclusion criteria were as follows: (1) histopathologically confirmed NSCLC; (2) PD-L1 expression assessed via immunohistochemical assays; (3) pretreatment CT imaging acquired within three months prior to the start of treatment; and (4) clear delineation of the primary tumor lesion on the CT image. The application of these inclusion criteria resulted in the selection of 482 patients in the primary dataset and 51 in the neoadjuvant cohort for further analysis (Figure 1). The primary cohort consisted of data from three academic institutions and was used for model development and primary testing, whereas the neoadjuvant cohort represented an independent validation set collected in a distinct clinical and temporal context. Our study adheres to the Declaration of Helsinki. The “CHUM Institutional Review Board”, through the Human Ethics Committee granted ethical approval for this study. Patients were either enrolled in the CHUM Lung Biobank and signed written informed consent forms or were included in project 18.255 (MP-02-2019-8091) at CHUM, which was approved by the CHUM Human Ethics Committee for retrospective analysis without direct patient contact.

For self-training purposes (refer to section: Self-training-based PD-L1 predictive pipeline), we included an additional 422 NSCLC patients from the NSCLC radiomics dataset [31], publicly available on The Cancer Imaging Archive [32]. The dataset consists of publicly available CT images, accompanied by segmentations delineating the primary lesions.

Clinical annotations included demographic information, treatment dates and types, the ECOG status and the smoking status as well as the clinical outcome of PFS at 6 months. Patient follow-up was conducted to document disease progression or death events for all study participants. The PFS was calculated starting from the surgical intervention date. The analysis excluded patients lacking data on their initial surgery. All patients involved in the study had a PFS greater than 6 months or experienced progression prior to this time point. In the three medical centers, PD-L1 expression was evaluated on diagnostic tissue specimens using IHC staining (Dako Autostainer) with the same 22C3 clone (pharmDx kit) and specimen type (biopsies). Timing of anti-PD-1 therapy relative to CT acquisition is reported in Table 1. PD-L1 expression in NSCLC tissue samples was measured using the Tumor Proportion Score (TPS). PD-L1 are reported by pathologists by either <1%, 1–49% and ≥ 50%. We compared our radiological PD-L1 reading to pathological PD-L1 high (>50%) vs. PD-L1 low (<1% and 1–49%).

### 2.2. CT Image Preparation and Pre-Processing

We pre-processed the pre-treatment CT images of NSCLC patients to ensure uniform data characteristics. Spatial resampling to isotropic 1 × 1 × 1 mm^3^ voxels was conducted to ensure uniform spacing for all images in the dataset, followed by truncation of Hounsfield Units (HU) to the lung window (window level = −600, window width = 1500). Subsequently, the primary lung tumor in each CT scan was segmented in the primary dataset and testing cohort of patients who underwent neoadjuvant treatment. For the primary dataset (used for training and primary testing), a semi-automatic segmentation of NSCLC was carried out as described in [29]. Primary lung tumors were annotated, and the lesions’ longest axes were identified by a radiation oncologist or radiologist. The segmentation process comprised three stages: (1) morphology-based chest isolation followed by chest segmentation using connected regions [33], (2) automatic lung segmentation using the detected boundary, and (3) intersection of the lung segmentation with a clustering-based lesion mask to identify lesion segmentation, taking lung symmetry into consideration. For the more recently recruited neoadjuvant cohort (used as a second hold-out test set), a recent fully automated deep learning segmentation algorithm was used to segment the NSCLC lesions [34] to account for the increased challenges in segmenting lesions with different stages. In total, 56,408 slices (from 1092 images) were used for training/internal validation and 20,843 slices (from 4823 images) were used for external validation. The pipeline performs lung isolation then segments the lesions within using an adapted U-Net architecture [35]. All segmentations were reviewed by an experienced radiation oncologist and were subject to manual adjustment when needed. This pre-processing pipeline ensured standardized image quality and accurate tumor delineation for subsequent analysis.

### 2.3. Radiomics Workflow

Radiomics features were extracted from each NSCLC lesion using the PyRadiomics v3.0.1 toolbox [36]. Prior to feature extraction, image filtering was performed using either a Laplacian of Gaussian (LoG) or Wavelet transformation. The LoG filter was applied to the original images with varying sigma values (0.1, 0.5, 1, 2, 3, 4, and 5), resulting in the generation of 651 LoG-derived features. In parallel, eight Wavelet decompositions were performed by applying high-pass or low-pass filters in each of the three dimensions, yielding a total of 744 Wavelet-derived features. The process resulted in a total of 1409 radiomics features, including 14 shape-based features, 270 first-order statistics, and 1125 texture-based features. To ensure consistency across features, standardization was applied to achieve a zero mean and unit standard deviation. Subsequently, dimensionality reduction was performed using the least absolute shrinkage and selection operator (LASSO) [37] fitted only on the training data. Within the training set, the LASSO regularization parameter was selected using 5-fold cross-validation restricted to the training partition. The resulting feature subset and coefficients were then fixed and applied unchanged to the internal test set and to the independent neoadjuvant cohort. No information from any test or validation data was used during feature selection, ensuring strict nesting and preventing data leakage.

### 2.4. Self-Training-Based PD-L1 Predictive Pipeline

**Figure 2:**
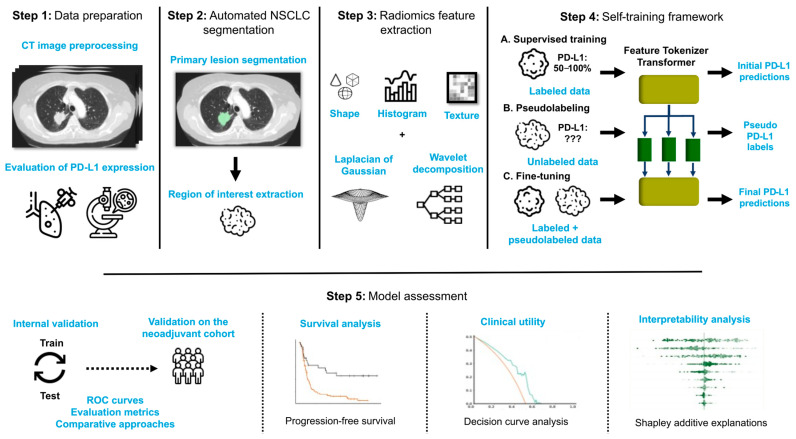
Overview of the AI-based PD-L1 classification pipeline. Pre-treatment CT images of patients with advanced NSCLC treated with ICIs are collected and pre-processed. The corresponding PD-L1 expression is associated and dichotomized into two classes: <50% vs. ≥50%. The ensuing images are segmented and radiomics features (shape, texture and histogram-based features) are extracted from the resulting lesions after the application of a Laplacian of Gaussian filter or a wavelet decomposition to the image. A self-training framework is then adopted in which the deep learning model, the Feature Tokenizer Transformer, is first trained using labeled data and then leveraged to generate pseudolabels for instances with no PD-L1 expression information. Labeled and pseudolabeled data are subsequently combined to fine tune the model and improve its predictive performance. The model is finally validated on the hold-out test set and a separate cohort of patients who underwent neoadjuvant treatment combining ICIs and chemotherapy.

The proposed AI-based pipeline combines the Feature Tokenizer Transformer (FT-Transformer) [38] with a self-training mechanism to predict whether NSCLC demonstrates a PD-L1 expression ≥ 50% (classified as PD-L1^High^) or less than 50% (classified as PD-L1^Low^), using radiomics features extracted from the segmented primary lesion on CT as input (Figure 2).

The FT-Transformer represents a specialized adaptation of the basic Transformer architecture, designed specifically for tabular data such as radiomics features. Its structure comprises three primary modules: the feature tokenizer, the Transformer encoder, and the classifier (Supplementary Figure S1).

To enhance the performance of the predictive pipeline and compensate for limited data, a self-training approach was designed for the FT-Transformer. First, the FT-Transformer was trained in a supervised manner utilizing solely the labeled dataset. This step produced initial predictions of marker expression, allowing for an evaluation of the pipeline’s performance. Subsequently, the trained pipeline was employed to generate pseudolabels for CT images lacking marker expression annotations, including both the public dataset and unlabeled in-house instances. Pseudolabeling was exclusively applied to instances for which the pipeline exhibited the highest confidence. This approach was motivated by the fact that the majority of erroneous predictions made by classifiers tend to occur in proximity to the decision boundary [39]. Consequently, pseudolabels were assigned only to instances for which the pipeline produced probabilistic outputs that fell outside the decision boundary. Following the pseudolabeling process, the pipeline underwent fine-tuning using both the originally labeled data and the newly pseudolabeled instances.

In order to prevent data leakage, pseudo-labels were generated exclusively for CT scans that lacked PD-L1 annotations and were not included in any training, internal testing, or external validation cohort. These unlabeled scans originated from two sources: (1) the publicly available NSCLC-Radiomics dataset from The Cancer Imaging Archive, which does not include PD-L1 measurements, and (2) in-house CT scans from NSCLC patients for whom PD-L1 immunohistochemistry was unavailable. Strict patient-level separation was enforced across all datasets using unique patient identifiers. No patient, scan, or image slice used for pseudo-labeling overlapped with any labeled training set, primary test set, or independent validation cohort. Pseudo-labels were generated only after the initial supervised training phase and were incorporated solely during model fine-tuning (Supplementary Table S1).

For training and primary validation, the primary dataset (N = 482) was randomly partitioned into a training subset (80%) and a hold-out test subset (20%), while ensuring that all splits were patient-based. This strategy was chosen to enable the training of a single final model for subsequent evaluation on the independent neoadjuvant cohort, while maintaining strict separation between development and validation. Given the substantial computational cost of the FT-Transformer self-training framework, nested or repeated cross-validation was not feasible in the present study. Pipeline training was conducted on an NVIDIA Titan RTX GPU equipped with 64 GB of RAM. For benchmarking purposes, the proposed pipeline’s performance was compared to the following machine learning and deep learning models, trained using the same data to perform the same PD-L1 classification task (≥50% vs. <50%): (1) extreme gradient boosting classifier (XGB), (2) Support Vector Machine (SVM), (3) random forest (RF), (4) Self-Attention and Intersample Attention Transformer (SAINT) [40]. Additionally, we evaluated the models aforementioned, which were trained using radiomics features, against two end-to-end models trained using voxel intensities directly: (1) the standard Vision Transformer (ViT) and (2) the Compact Convolutional Transformer (CCT) [41], designed specifically for small datasets. For these two models, 3D volumes of NSCLC tumors were resized to 50 × 50 × 50, and voxel intensities were normalized to achieve a zero mean and unit standard deviation. To mitigate overfitting, data augmentation was applied through random horizontal and vertical flipping during training. The binary cross-entropy loss function and the Adam optimizer were utilized to train all deep learning models. Given the substantial computational cost of the FT-Transformer self-training framework, extensive hyperparameter optimization was not performed. Instead, all models were trained using fixed hyperparameter configurations selected based on prior literature and preliminary feasibility experiments performed without access to the test sets (Supplementary Table S2). Model calibration was assessed using calibration curves and the Brier score. In addition, post hoc recalibration using isotonic regression was explored.

### 2.5. Interpretability of the AI-Based Pipeline

We conducted a comprehensive analysis of the pipeline’s behavior by applying the Shapley Additive Explanations (SHAP) technique to the produced predictions [42], for both the primary and neoadjuvant treatment regimen cohort. SHAP is a technique that calculates Shapley values for each feature, representing their individual contributions to the pipeline’s output. We then ranked features based on their average Shapley values across all instances. Positive Shapley values suggest that a feature influences the pipeline towards predicting high scores, while negative values indicate a tendency towards low score predictions.

### 2.6. Statistical Analysis

Statistical analysis was performed using non-parametric methods. For comparisons of numerical variables between groups, the Wilcoxon rank sum test was employed. For categorical variables, Fisher’s exact test was utilized. Decision curve analysis was performed to evaluate the potential net benefit of using the rad-PDL1 score to support patient stratification based on predicted PD-L1 status, compared with treat-all and treat-none strategies, across a range of clinically plausible probability thresholds. The analysis was plotted using Python dcurves v1.1.0 package. For each threshold probability, the net benefit was calculated. Net benefit was computed as a weighted combination of true positives and false positives, where the weight is derived from the threshold probability. Specifically, it represents the proportion of patients correctly identified for treatment minus the proportion incorrectly identified, adjusted by the threshold probability. Kaplan–Meier survival curves were produced using the Python Lifelines v0.27.8 library, with statistical comparisons conducted using the log-rank test. To perform survival analysis, we derived the FT-Transformer class probabilities, denoted as the rad-PDL1 score. These probabilities were then categorized into rad-PDL1^High^ and rad-PDL1^Low^ groups. The rad-PD-L1 cutoff was defined on the training cohort only using X-tile, fixed, and applied unchanged to the test set to avoid optimistic bias. Survival analyses in the neoadjuvant cohort were considered exploratory due to the limited number of events. To assess the independent prognostic value of the rad-PDL1 score and other clinical characteristics simultaneously, we carried out a multivariate Cox proportional hazards regression analysis. This method generated hazard ratios (HR) along with their corresponding 95% confidence intervals. Statistical significance was defined as a two-sided *p*-value less than 0.05.

## 3. Results

### 3.1. Patient Cohort

The clinicopathological properties of the NSCLC patients for both the primary cohort (N = 482 patients) and the cohort of patients who underwent neoadjuvant treatment (N = 51 patients) are detailed in Table 1. In the primary cohort, the median age was 66 (interquartile range: 61–70), with a roughly equal representation of men (51%) and women (49%). In the neoadjuvant cohort, the median age was 68 (61–73), with a predominance of male patients (55%). Active smokers comprised 29% and 27% of patients in the primary testing and neoadjuvant cohorts, respectively. Approximately half of patients had a PD-L1 expression ≥ 50% in the primary testing set, compared to 59% in the neoadjuvant cohort. The proportion of patients with a PFS greater than 6 months was 54% and 82% in the primary testing set and the neoadjuvant cohorts, respectively. The median PFS was 6.6 months in the primary cohort and 15.2 months in the neoadjuvant cohort. Upon statistical evaluation, the aforementioned features showed no significant differences between the two cohorts, except for the PFS (*p* = 0.0001) and the ECOG status (0.020).

### 3.2. Prediction of PD-L1 Expression and Validation on the Neoadjuvant Cohort

**Figure 3:**
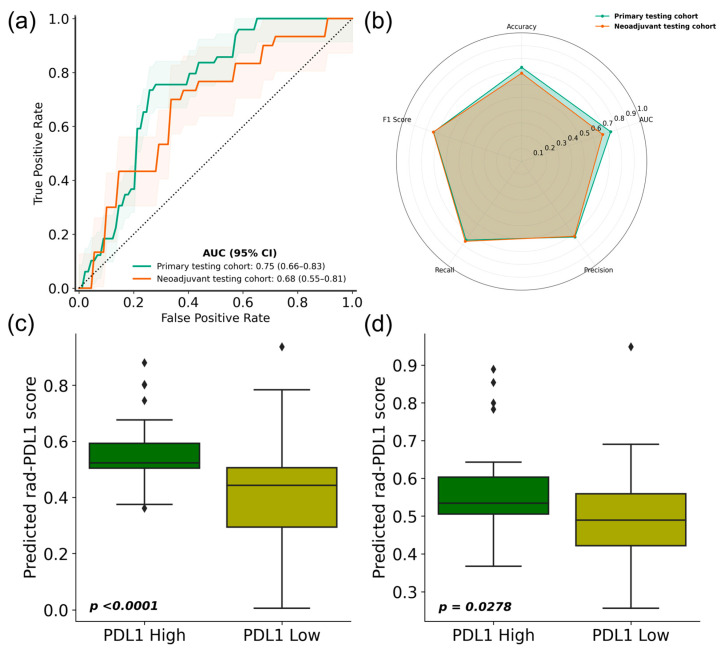
PD-L1 classification results of the predictive pipeline. (**a**) Receiver operating characteristics curve on the primary testing dataset (green) and the validation cohort consisting of patients who underwent neoadjuvant treatment (orange). (**b**) Spider plot showing the performance metrics of the predictive pipeline on the primary and neoadjuvant validation cohorts. AUC: area under the receiver operating characteristic curve. (**c**,**d**) Evaluation of the probabilistic rad-PDL1 score obtained using the predictive pipeline. The box plots present the distribution of rad-PDL1 by PD-L1 expression level (high vs. low). The data indicated a statistically significant difference in rad-PDL1 between the PD-L1^High^ and PD-L1^Low^ groups in both (**c**) the primary testing cohort (Mann–Whitney U test; *p* < 0.0001) and (**d**) the neoadjuvant testing cohort (*p* = 0.0298).

**Figure 4:**
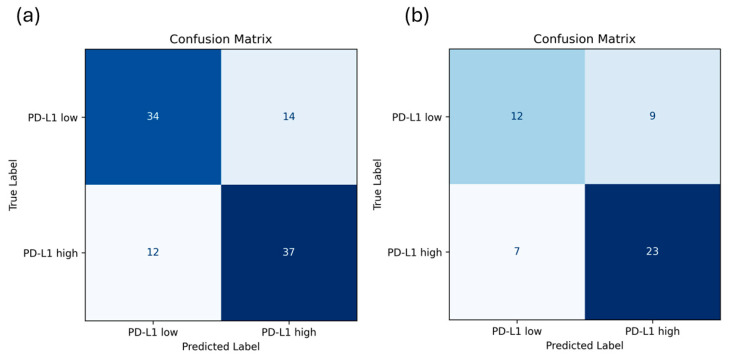
Confusion matrices of the model evaluated on (**a**) the primary test set and (**b**) the independent neoadjuvant cohort.

We assessed the prediction performance of the AI-based pipeline on the primary testing set of the primary cohort and its generalizability on the cohort of patients who underwent the neoadjuvant treatment regimen by comparing the pipeline predictions with the expression values obtained by IHC. In the primary testing set of the primary cohort, the proposed pipeline’s ability to distinguish between rad-PDL1^High^ and rad-PDL1^Low^ was demonstrated by achieving an AUC of 0.75 (95% confidence interval: 0.66–0.83). The accuracy, precision, recall and F1-score were 0.73 (0.64–0.82), 0.73 (0.64–0.81), 0.76 (0.67–0.84) and 0.74 (0.65–0.83), respectively. Furthermore, the pipeline reached an AUC of 0.68 (95% CI 0.55–0.81) on the neoadjuvant testing cohort, comprising patients receiving neoadjuvant treatment. The accuracy, precision, recall and F1-score were 0.69 (0.56–0.81), 0.72 (0.60–0.84), 0.77 (0.65–0.88) and 0.74 (0.62–0.86), respectively (Figure 3, Supplementary Table S3). As expected, model performance decreased in the neoadjuvant cohort, reflecting differences in disease stage, treatment strategy, and outcome distribution relative to the training population. The neoadjuvant cohort was not used during model training and therefore constitutes an independent external validation rather than a site-stratified test set. The confusion matrices of the model evaluated on the primary test set and the independent neoadjuvant cohort are shown in Figure 4. Using an operating point selected to target a sensitivity of about 0.7, the model demonstrated a specificity of 0.75 and 0.67 on the primary test set and the independent neoadjuvant test set, respectively.

There was a statistically significant difference in the predicted rad-PDL1 score between the PDL1^High^ and PDL1^Low^ tumors in both the primary testing cohort (Mann–Whitney U test; *p* < 0.0001) and the neoadjuvant cohort (*p* = 0.0298). The standardized effect size in rad-PDL1 scores between PDL1^High^ and PDL1^Low^ tumors was large (Cohen’s d = 0.91), indicating substantial separation beyond visual boxplot differences. Moreover, the predictive performance of the self-training pipeline leveraging the FT-Transformer was compared to other deep and conventional machine learning models. The FT-Transformer trained using a self-training framework outperformed deep and machine learning models in PD-L1 classification. The models trained using radiomics features outperformed the end-to-end deep learning models, namely the ViT and the CCT. Comparative benchmarking of radiomics-based models, including FT-Transformer variants, is shown in Supplementary Figure S2. Calibration curves and Brier scores are shown in Supplementary Figure S3. The rad-PDL1 score demonstrated reasonable calibration in the primary test set (Brier score = 0.208), with slightly increased uncertainty observed in the neoadjuvant cohort, consistent with its sample size (Brier score = 0.229). Exploratory recalibration modestly improved probability alignment (Brier score = 0.172 and 0.213 on the primary and neoadjuvant test sets, respectively).

### 3.3. Interpretability Analysis

**Figure 5:**
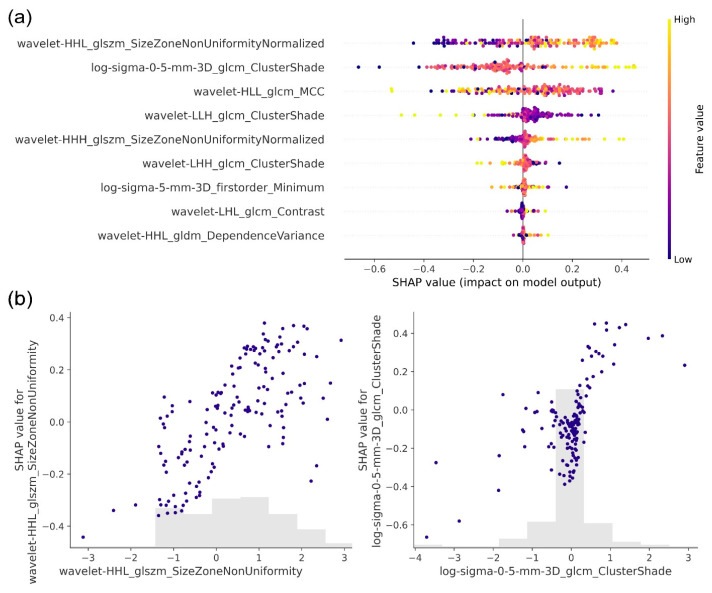
Interpretability analysis of the predictive pipeline. (**a**) Summary plot showing the top features obtained using the Shapley Additive Explanations (SHAP) technique, according to their average Shapley values, which reflect their impact on the model’s output. The top six features were found to be textural features, which highlights the importance of texture-related properties of NSCLC lesions in classifying NSCLC. (**b**) Variation in SHAP values of the top two radiomics features as a function of their actual values. The results show that heterogeneous NSCLC lesions were associated with a high PD-L1 expression. Every point represents a testing set instance and the gray area depicts the distribution of the corresponding SHAP values.

After training and evaluating the PD-L1 prediction pipeline, we sought to interpret the pipeline’s predictions by evaluating the relative contribution of each radiomics feature using the SHAP technique. Figure 5 shows the top-ranking radiomics features according to their average Shapley values, which reflect their impact on the pipeline’s output. The top six features were exclusively textural, emphasizing the significance of the textural properties of NSCLC lesions in classifying PD-L1 expression.

The top feature returned by the SHAP analysis was the Size Zone Non-Uniformity Normalized (SZNUN) extracted after the application of a wavelet decomposition using high-pass filters along the x and y-axes and a low-pass filter along the z-axis (Wavelet-HHL). The Size Zone Non-Uniformity Normalized (SZNUN) measures the variability of size zone volumes in the lung lesion, normalized by the square of the total number of zones. A lower SZNUN value indicates greater homogeneity among the size zone volumes in the image. The SHAP analysis revealed that higher SZNUN values corresponded to positive Shapley values, influencing the pipeline to predict elevated PD-L1 expression levels. This suggests that increased heterogeneity in size zone volumes within the image is associated with higher predicted marker expression according to the model, reflecting feature attribution rather than biological causation. The second top-ranking radiomics feature was ClusterShade, extracted after the application of a LoG filter (sigma value = 0.5 mm) to the image. The Clustershade feature encodes the skewness or asymmetry in the image texture. Low ClusterShade values suggest more symmetry and uniformity in the texture. The latter feature demonstrated a pattern comparable to the SZNUN, where elevated feature values corresponded to positive Shapley values. Consequently, this relationship led to predictions of high PD-L1 expression scores by the pipeline. These results indicate that the model assigns higher predicted rad-PDL1 scores to lesions exhibiting more intricate or heterogeneous textural patterns. SHAP feature rankings were highly consistent across test partitions, with a mean Spearman rank correlation of 0.95 ± 0.03. Notably, the top five features were identical across all three partitions, indicating robust and stable model attribution. The consistency of feature rankings is visualized in Supplementary Figure S4.

**Figure 6:**
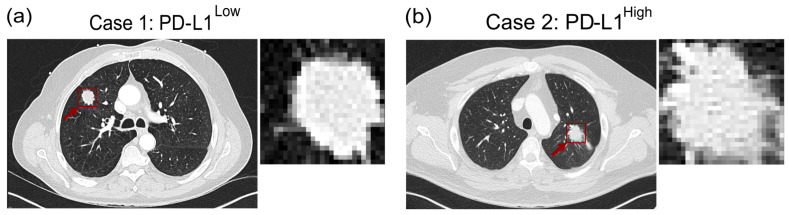
Representative cases with low and high PD-L1 expressions. (**a**) A pretreatment CT image of a PD-L1^High^ NSCLC (arrow) in the right lung. (**b**) A pretreatment CT image of a PD-L1^Low^ NSCLC (arrow) in the left lung.

In line with these findings, Figure 6 shows representative cases of NSCLC tumors with low and high PD-L1 expressions, respectively. The pretreatment CT image in Figure 6a demonstrates an NSCLC in the right lung with a high PD-L1 expression (TPS: 50–100%), as evidenced by IHC. Correspondingly, the pipeline classified it as a rad-PDL1^High^ NSCLC. In contrast, the NSCLC observed in the CT image of Figure 6b has a low PD-L1 expression (TPS < 50%) and was classified as rad-PDL1^Low^. These cases confirm the hypothesis that distinct CT features can be exhibited between PD-L1^High^ and PD-L1^Low^ NSCLC. This observation suggests a potential correlation between PD-L1 expression levels and radiographic characteristics of NSCLC, which has potential implications for non-invasive tumor assessment and treatment planning.

### 3.4. Decision Curve Analysis

**Figure 7:**
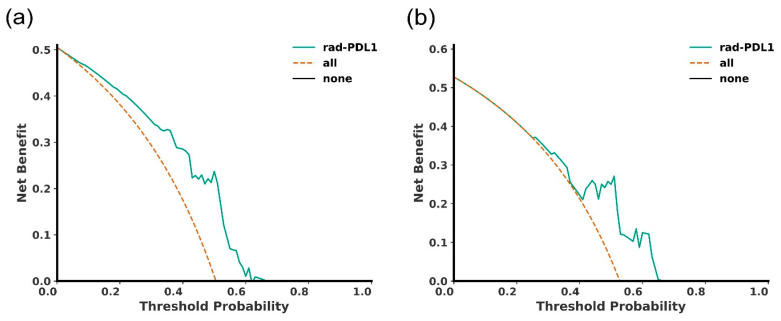
Decision curve analysis by modality on (**a**) the primary and (**b**) the neoadjuvant testing cohorts. The net benefit is computed for each threshold probability, representing the acceptable risk beyond which acting (treatment, further testing, etc.) is advised. The dashed orange curve represents the approach where all patients are considered to have a high expression of PD-L1 and the black x-axis represents the approach where no patient is considered to have a high PD-L1 expression. As shown in the plots, the net benefit of the proposed pipeline (green curve) is higher than the “treat all patients as high PD-L1” (orange curve) and “treat no patient as high PD-L1” (black line) approaches in both the primary and neoadjuvant testing cohorts.

Across probability thresholds of 0.25–0.68, the proposed model demonstrated a positive net benefit (green curve) relative to treat-all (orange line) and treat-none (black x-axis) strategies, suggesting potential utility for patient stratification in exploratory contexts (Figure 7). This probability range corresponds to thresholds at which a clinician might reasonably favor additional confirmatory testing over default treat-all or treat-none strategies, consistent with the intended exploratory use of the model.

### 3.5. Association of Rad-PDL1 with Clinical Outcomes

**Figure 8:**
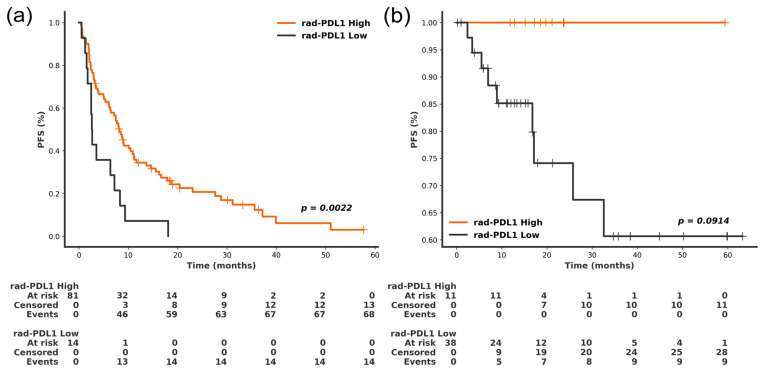
Prognostic significance of the predicted rad-PDL1 score in (**a**) the primary testing cohort and (**b**) the neoadjuvant testing cohort composed of patients receiving neoadjuvant treatment. The progression-free survival was significantly shorter in the rad-PDL1^Low^ group compared to the rad-PDL1^High^ in the primary testing cohort (*p* = 0.02). Vertical lines represent censored observations.

We finally evaluated the potential clinical impact of categorizing patients based on high versus low expression levels of rad-PDL1 (Figure 8). Consistent with the improved prognosis observed in patients with high PD-L1 expression treated with ICIs [43,44,45], patients with rad-PDL1^High^ NSCLC had a longer median PFS than patients with rad-PDL1^High^ NSCLC in the primary testing set (8.19 vs. 2.69 months; *p* = 0.002). In the neoadjuvant cohort, the median PFS in the rad-PDL1^High^ vs. rad-PDL1^Low^ groups was 18.6 vs. 13.8 months (*p* = 0.09). Given the limited number of observed events, analyses in the neoadjuvant cohort are considered exploratory. The incidence of pathologic complete response in the neoadjuvant cohort was 45.5% and 17.9% in the rad-PDL1^High^ and the rad-PDL1^Low^ groups, respectively (*p* = 0.10). The median OS was 15.2 vs. 12.0 months and 18.6 vs. 14.8 months in rad-PDL1^High^ vs. rad-PDL1^Low^ groups, in the primary and neoadjuvant cohort respectively (*p* > 0.05). The multivariate analysis (Table 2) confirmed that the prognostic significance of the rad-PDL1 marker for PFS was independent of the clinicopathological characteristics (*p* = 0.01). No violations were detected for the covariates included in the multivariable model.

## 4. Discussion

The PD-L1 expression, assessed via IHC, is currently the only approved biomarker by the Food and Drug Administration (FDA) for guiding ICI therapy in advanced NSCLC [29]. It is also the most well-established and widely utilized biomarker for ICIs in routine clinical practice [46]. Positive PD-L1 expression was linked to better outcomes and higher clinical response rates compared to patients with negative PD-L1 expression [47]. In this study, we proposed an AI-based pipeline based on self-training, Transformer networks and radiomics features extracted from pretreatment CT, to classify PD-L1 expression in NSCLC amenable to ICIs. We also validated the pipeline on a cohort of patients with limited NSCLC treated with a neoadjuvant regimen combining ICIs and chemotherapy. Although the proposed pipeline demonstrated robust performance in the primary multi-institutional cohort, performance was attenuated in the independent neoadjuvant cohort. This reduction likely reflects fundamental differences in disease stage, treatment regimen, and biological behavior, rather than model failure. Nevertheless, the model retained discriminatory power, supporting the hypothesis that CT-derived radiomic features encode biologically meaningful information related to PD-L1 expression, albeit with reduced effect size in a distinct clinical context.

Furthermore, the potential clinical relevance of the rad-PDL1 score derived from the pipeline was corroborated by its association with oncological outcomes, demonstrating its potential prognostic value. Model performance remained consistent across centers (AUC range 0.69–0.83), and inclusion of center as a covariate in multivariable models did not materially alter the observed associations. The impact of clinicopathological factors was primarily assessed through multivariable modeling rather than subgroup analyses to avoid underpowered stratifications. Its potential utility in the clinical setting was evaluated by the decision curve analysis results obtained in both cohorts. Importantly, the observed net benefit reflects potential value in supporting stratification decisions prior to or alongside tissue-based assessment, rather than replacing pathological testing, and should be interpreted in the context of the model’s moderate external performance.

The noninvasive assessment of PD-L1 expression using CT images provides several advantages over traditional IHC-based methods, based on biopsies. Notably, the high accessibility and cost-effectiveness of this approach make it a valuable tool for dynamically monitoring treatment responses. This is particularly relevant in NSCLC, as it enables longitudinal studies to repeatedly assess PD-L1 expression through follow-up scans, facilitating the detection of acquired resistance to ICIs during treatment. Such insights could guide timely adjustments to therapeutic strategies, improving patient outcomes [5,48]. Moreover, the noninvasive approach can be leveraged to evaluate spatial heterogeneity between the primary tumor and metastases. Metastatic lesions may exhibit disparate PD-L1 positivity rates, which can impact treatment decisions and response to ICIs [49,50]. Additionally, understanding the variation in PD-L1 expression profiles may assist physicians in selecting optimal biopsy locations [51], thus reducing false-negatives.

This work leverages advanced deep learning techniques, including self-training and the feature tokenizer Transformer, which improves the classification accuracy and generalizability of the AI-based pipeline. In computer vision applications, Transformers excel at focusing on key image regions, but their data requirements and susceptibility to overfitting make them challenging with limited data, especially in the high-dimensional space of CT images. Radiomics provides a valuable alternative to end-to-end pipelines by reducing the feature space and extracting more well-established interpretable features. Furthermore, this allowed us to conduct a post hoc analysis by employing the SHAP method, allowing for a detailed investigation into the pipeline’s decision-making process. High rad-PDL1 scores were associated with intratumoral textural heterogeneity in NSCLC. These findings are concordant with previous work showing that high gray level co-occurrence matrix entropy, a feature encoding textural randomness and irregularity, was correlated with high PD-L1 expression scores [52]. From a radiographic perspective, higher SZNUN values, which were consistently associated with positive SHAP values, correspond to lesions exhibiting greater internal heterogeneity, potentially reflecting mixed attenuation patterns, irregular internal architecture, or necrotic components on CT. Similarly, higher ClusterShade values indicate skewed and asymmetric texture patterns, which may correspond to non-uniform tumor density, abrupt transitions between solid and less dense regions, or irregular margins. Beyond these two dominant features, the rad-PD-L1 score integrates multiple complementary texture-based descriptors capturing spatial complexity, asymmetry, and variability in voxel intensity distributions. Collectively, these features suggest that tumors predicted to have high PD-L1 expression tend to display increased intratumoral complexity rather than uniform solid morphology (Supplementary Table S4). Importantly, these associations represent model attribution patterns rather than causal biological relationships.

Nevertheless, this work presents some limitations. First, while the multi-institutional dataset includes patients treated at three distinct medical centers and utilizes a variety of scanners, including those from Siemens, GE Medical Systems, Philips, and Toshiba, all hospitals are located within the same province in Canada with a relatively limited number of patients. To achieve a more epidemiologically diverse dataset, large-scale prospective multi-center studies involving patients from different regions in the world may be required. Second, the work relies solely on CT scans. Multimodal approaches involving different imaging modalities and data sources might boost the performance of our predictive pipeline further. In fact, recent studies have demonstrated the association between positron emission tomography (PET) features and PD-L1 expression in NSCLC [53,54]. Consequently, integrating PET and CT features using efficient fusion techniques may prove beneficial in this context. Moreover, the two imaging modalities could be combined with other data types to develop an integrative and reliable survival pipeline. Third, the interval between pretreatment CT imaging and histopathological analysis varied among patients, potentially allowing for temporal fluctuations in PD-L1 expression due to its dynamic nature. To mitigate this issue, we restricted our study to CT scans acquired within a three-month window prior to treatment initiation. This approach aims to minimize the impact of temporal heterogeneity on the assessment of PD-L1 expression and its relationship to imaging features. Furthermore, while nested or repeated cross-validation would provide more robust estimates of performance variability, such approaches were constrained by the computational demands of the self-training FT-Transformer framework and the need to define a single model for independent validation. Future studies leveraging larger datasets and increased computational resources should incorporate repeated or nested cross-validation to further strengthen model selection and uncertainty quantification. Finally, while leave-one-center-out validation would further isolate site-specific effects, such analyses were limited by center-wise sample size after PD-L1 stratification. Future large-scale prospective studies will be required to support robust leave-one-center-out evaluation. Our findings are currently supported in two specific clinical contexts: advanced-stage NSCLC treated with ICI and an exploratory limited-stage neoadjuvant cohort treated with chemo-immunotherapy. The applicability of the proposed radiomic surrogate to other disease stages, non-ICI treatment regimens, or institutions not represented in the present datasets remains to be established. Accordingly, the current results should not be extrapolated beyond these validated settings without further prospective multi-site evaluation.

## 5. Conclusions

In summary, this study presents a multi-institutionally trained, CT-based imaging surrogate of PD-L1 expression that demonstrates feasibility across distinct NSCLC populations. While exploratory external validation in a neoadjuvant cohort showed promising but limited performance, the preservation of discriminatory power supports the biological relevance of the proposed approach. At present, validation is limited to ICI-treated advanced-stage NSCLC and an exploratory neoadjuvant cohort, and additional studies are warranted to assess generalizability to other clinical settings. Further large-scale, prospective, and cohort-specific validation will be required before clinical implementation to validate whether the model can (1) select suitable candidates for ICIs; (2) noninvasively assess PD-L1 expression in patients with limited NSCLC amenable to neoadjuvant chemo-ICIs; (3) evaluate primary and acquired resistance to ICIs; (4) identify patients who might benefit from a closer follow-up or a potential treatment modification; and (5) understand spatial and temporal heterogeneity of the tumor microenvironment. In future investigations, our proposed AI-based pipeline could be evaluated and potentially adapted for the prediction of PD-L1 expression in other malignancies where it has received FDA approval for guiding ICIs such as bladder, cervical and triple-negative breast cancer. In addition, a comprehensive international database could be leveraged to develop a multimodal approach to reliably predict PD-L1 expression and prognosis of patients with disparate epidemiological characteristics worldwide. While clinical variables are likely to provide complementary information, their integration with radiomics was beyond the scope of this study and will be explored in future work.

## Figures and Tables

**Table 1:** Clinicopathological characteristics of the primary and neoadjuvant cohorts.

	Primary Cohort (N = 482)	Neoadjuvant Cohort (N = 51)	*p*-Value
**Age**	66 (61–70)	68 (61–73)	0.167
**Sex**-n (%)			0.659
Male	244 (51)	28 (55)	
Female	238 (49)	23 (45)	
**Smoking status**-n (%)			0.869
Current	139 (29)	13 (27)	
Former or never	338 (71)	36 (73)	
**ECOG status**-n (%)			0.020
>1	67 (14)	1 (2)	
≤1	415 (86)	46 (98)	
**PD-L1 status**-n (%)			0.303
≥50%	243 (50)	30 (59)	
<50%	239 (50)	21 (41)	
**Progression-free survival**-n (%)			0.0001
≥6 months	257 (54)	41 (82)	
<6 months	220 (46)	9 (18)	
**Duration between CT and anti-PD-1**	1.31 (0.59–2.41)	1.64 (0.69–2.52)	0.531
**Slice thickness**	2.0 (2.0–2.0)	2.0 (2.0–2.0)	0.187
**Pixel spacing**	0.70 (0.65–0.76)	0.75 (0.69–0.78)	0.0005
**CT scan KVP**	120 (100–120)	100 (100–120)	0.003

**Table 2:** Multivariate analysis of progression-free survival (PFS) according to the rad-PDL1 score and clinical features. The rad-PDL1 score was a predictor of the PFS, independently of the clinical features.

	Progression-Free Survival	
	HR (95% CI)	*p*-Value
**rad-PDL1 (<50% vs. ≥50%)**	0.39 (0.20–0.76)	0.01
**Age (<60 vs. ≥60)**	0.66 (0.37–1.20)	0.17
**Sex (female vs. male)**	1.23 (0.75–2.02)	0.41
**Current smoker (no vs. yes)**	0.81 (0.48–1.36)	0.43
**ECOG (<2 vs. ≥2)**	1.44 (0.67–3.11)	0.35
**Treatment regimen (IO first line vs. not)**	1.60 (0.97–2.66)	0.07
**Histology (adenocarcinoma vs. other)**	1.39 (0.74–2.61)	0.3
**Site (medical center)**	1.19 (0.85–1.67)	0.31
**Stage (IV vs. other)**	1.08 (0.65–1.79)	0.77

## Data Availability

Imaging data and associated clinicopathological characteristics and marker quantification are available upon request from the authors.

## Supplementary Materials

The following supporting information can be downloaded at: https://www.mdpi.com/article/10.3390/cancers18040552/s1, Figure S1: Architecture of the Feature-Tokenizer Transformer (FT-Transformer) leveraged in this study. Figure S2: Comparison of the performance of different deep learning and machine learning models on the PD-L1 classification task (<50% vs. ≥50%). Figure S3: Calibration curves of the FT-Transformer model. Figure S4: SHAP feature rank stability across test partitions. Table S1: Data Leakage Audit Checklist. Table S2: Summary of model inputs, augmentation strategies, and hyperparameter configurations. Table S3: Performance metrics of the proposed predictive pipeline on the primary and neoadjuvant validation cohorts. Table S4: Key radiomic features contributing to the rad-PD-L1 score and their radiologic interpretation.
