# Supplementary material for: Multi-Institutional CT Scan-Based Radiomics for Predicting Tumor PD-L1 Expression in Patients with Advanced and Limited Non-Small Cell Lung Cancer
Source: Cancers (Basel). 2026 Feb 8;18(4):552. doi: 10.3390/cancers18040552 (PMC12939105; doi:10.3390/cancers18040552)
Supplement: Supplementary file 1 [file cancers-18-00552-s001.zip › cancers-4107804-supplementary.pdf]

Supplementary Material

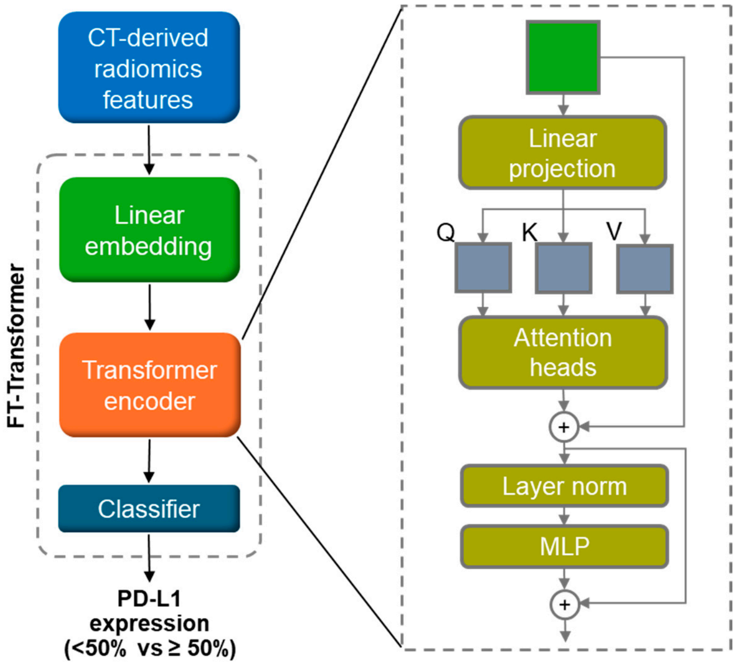

Supplementary Figure S1. Architecture of the Feature-Tokenizer Transformer (FT-Transformer) leveraged in this study. The FT-Transformer adapts the Transformer architecture for tabular data, consisting of three key components: feature tokenizer, Transformer encoder, and classifier. The feature tokenizer is responsible for transforming each input feature into an embedding through a linear transformation. Subsequently, the embeddings of all features are concatenated and augmented with a classification token. This combined representation serves as an input for the subsequent stages of the model. The resulting representation is passed through the Transformer encoder, which consists of multi-head self-attention and feed-forward modules. The final classification is then carried out by applying a fully connected layer to the classification token to produce the output (PD-L1 expression <50% vs ≥50%). The model had 2 attention heads, an embedding dimension of 32 and was trained for 20 epochs.

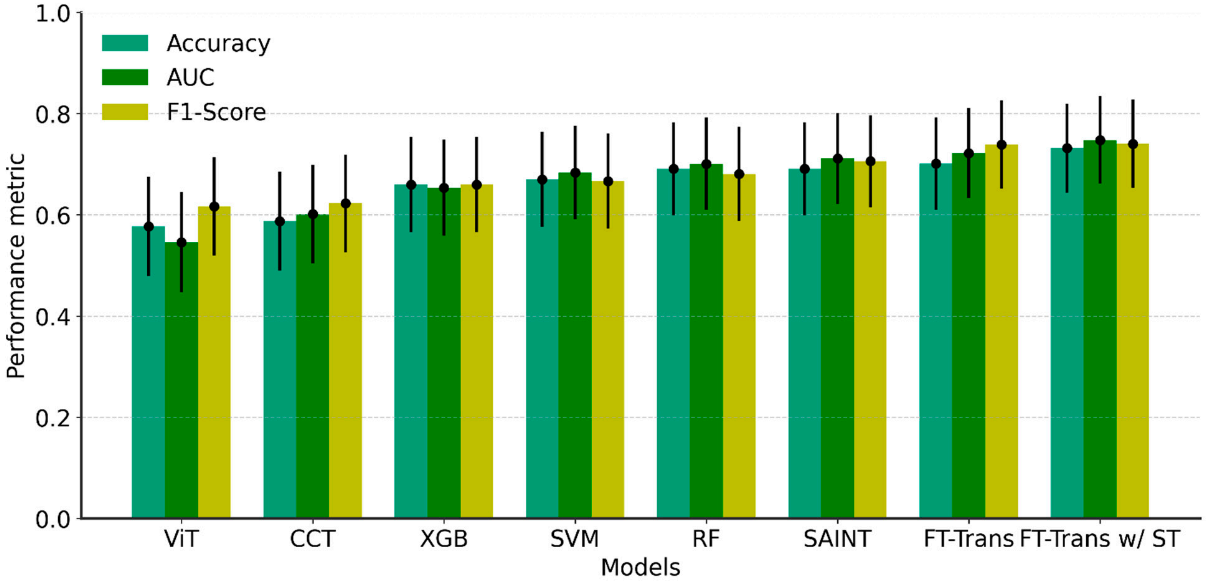

Supplementary Figure S2. Comparison of the performance of different deep learning and machine learning models on the PD-L1 classification task (<50%vs ≥50%). The FT-Transformer model trained using a self-training scheme (FT-Trans w/ ST) outperformed all the comparative models. Moreover, the models trained using radiomics features outperformed those trained end-to-end, namely the ViT and the CCT. Error bars represent 95% confidence intervals.

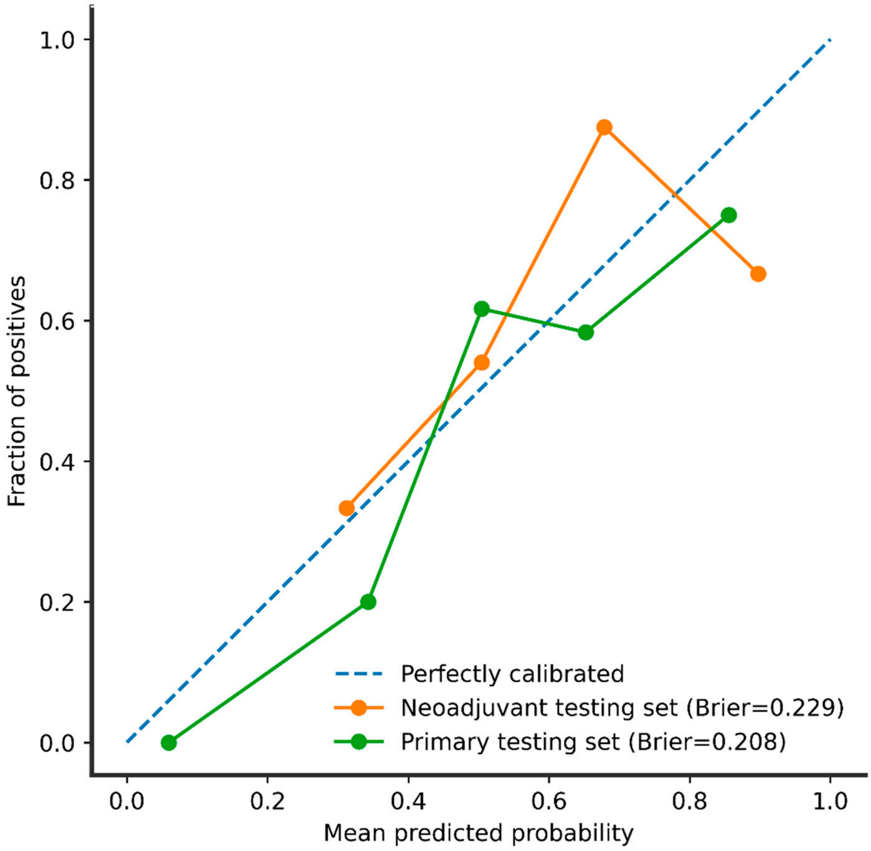

Supplementary Figure S3. Calibration curves of the FT-Transformer model on the primary test set and the independent neoadjuvant test cohort.

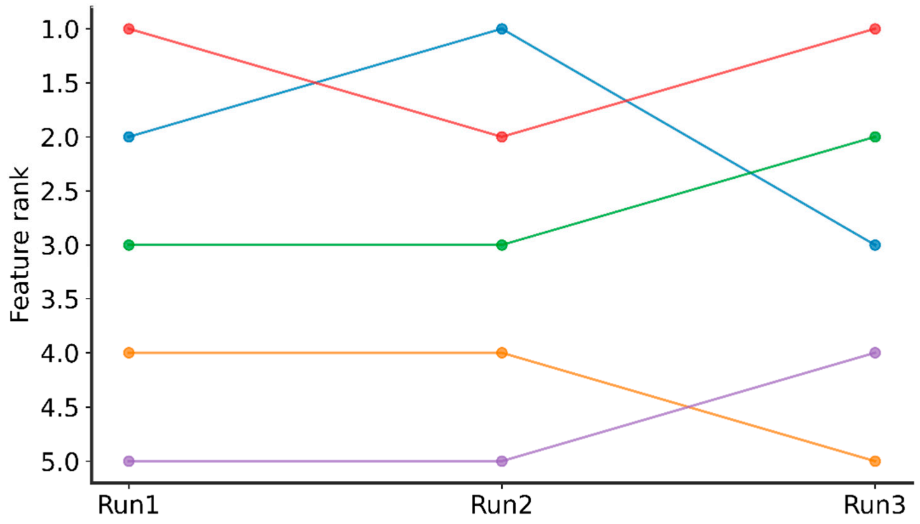

Supplementary Figure S4. SHAP feature rank stability across test partitions.

Supplementary Table S1. Data Leakage Audit Checklist

Audit item	Confirmed
Patient-level identifiers used for all splits	✓
No patient overlap between labeled training and primary test sets	✓
No patient overlap between labeled data and pseudo-labeled data	✓
No scan or slice overlap across any cohorts	✓
Pseudo-labeling applied only to unlabeled data	✓
Independent (neoadjuvant) cohort excluded from all training and pseudo-labeling steps	✓
Pseudo-labels generated after initial supervised training	✓
Test labels never used for pseudo-label generation	✓

Supplementary Table S2. Summary of model inputs, augmentation strategies, and hyperparameter configurations

Model	Input modality	Input representation	Data augmentation	Hyperparameter strategy
**ViT**	CT images	3D image patches	Random flips	Fixed configuration
**CCT**	CT images	3D image patches	Random flips	Fixed configuration
**XGB**	Radiomics	Radiomic features	None	Fixed configuration
**SVM**	Radiomics	Radiomic features	None	Fixed configuration
**RF**	Radiomics	Radiomic features	None	Fixed configuration
**SAINT**	Radiomics	Radiomic features	None	Fixed configuration
**FT-Trans**	Radiomics	Radiomic features	None	Fixed configuration
**FT-Trans w/ ST**	Radiomics	Radiomic features	None	Fixed configuration

Supplementary Table S3. Performance metrics of the proposed predictive pipeline on the primary and neoadjuvant validation cohorts. 95% confidence intervals are presented between parentheses.

Model	Accuracy	AUC	Precision	Recall	F1-score
**Primary test set**	0.73 (0.64-0.82)	0.75 (0.66-0.83)	0.73 (0.64-0.81)	0.76 (0.67-0.84)	0.74 (0.65-0.83)
**Neoadjuvant cohort**	0.69 (0.56-0.81)	0.68 (0.55-0.81)	0.72 (0.60-0.84)	0.77 (0.65-0.88)	0.74 (0.62-0.86)

Supplementary Table S4. Key radiomic features contributing to the rad-PD-L1 score and their radiologic interpretation. The features are ranked according to their SHAP score from top to bottom. GLSZM: Gray Level Size Zone Matrix; GLCM: Gray Level Co-occurrence Matrix; GLDM: Gray Level Dependence Matrix.

Radiomic feature	Radiomics family	Image filter	Radiologic interpretation
wavelet-HHL_glszm_SizeZoneNonUniformityNormalized	GLSZM	Wavelet (HHL)	Increased intratumoral heterogeneity with variable-sized homogeneous regions.
log-sigma-0-5-mm-3D_glcm_ClusterShade	GLCM	LoG (σ = 0.5 mm)	Asymmetric internal texture with patchy or skewed density patterns.
wavelet-HLL_glcm_MCC	GLCM	Wavelet (HLL)	High internal texture complexity and voxel interdependence.
wavelet-LLH_glcm_ClusterShade	GLCM	Wavelet (LLH)	Non-uniform and asymmetric internal density distribution.
wavelet-HHH_glszm_SizeZoneNonUniformityNormalized	GLSZM	Wavelet (HHH)	Fine-scale textural heterogeneity across the lesion.
wavelet-LHH_glcm_ClusterShade	GLCM	Wavelet (LHH)	Abrupt transitions between denser and less dense regions.
log-sigma-5-mm-3D_firstorder_Minimum	First-order	LoG (σ = 5 mm)	Presence of low-attenuation components within the tumor.
wavelet-LHL_glcm_Contrast	GLCM	Wavelet (LHL)	Marked local intensity differences between adjacent regions.
wavelet-HHL_gldm_DependenceVariance	GLDM	Wavelet (HHL)	Irregular spatial organization of voxel dependencies.
